# Imbalanced Text Sentiment Classification Based on Multi-Channel BLTCN-BLSTM Self-Attention

**DOI:** 10.3390/s23042257

**Published:** 2023-02-17

**Authors:** Tiantian Cai, Xinsheng Zhang

**Affiliations:** 1 School of Information and Control Engineering, Xi’an University of Architecture and Technology, Xi’an 710055, China; 2 School of Management, Xi’an University of Architecture and Technology, Xi’an 710055, China

**Keywords:** multi-channel BLTCN-BLSTM, imbalanced short text, self-attention, loss rebalancing, enhancement of classifier

## Abstract

With the continuous expansion of the field of natural language processing, researchers have found that there is a phenomenon of imbalanced data distribution in some practical problems, and the excellent performance of most methods is based on the assumption that the samples in the dataset are data balanced. Therefore, the imbalanced data classification problem has gradually become a problem that needs to be studied. Aiming at the sentiment information mining of an imbalanced short text review dataset, this paper proposed a fusion multi-channel BLTCN-BLSTM self-attention sentiment classification method. By building a multi-channel BLTCN-BLSTM self-attention network model, the sample after word embedding processing is used as the input of the multi-channel, and after fully extracting features, the self-attention mechanism is fused to strengthen the sentiment to further fully extract text features. At the same time, focus loss rebalancing and classifier enhancement are combined to realize text sentiment predictions. The experimental results show that the optimal F1 value is up to 0.893 on the Chnsenticorp-HPL-10,000 corpus. The comparison and ablation of experimental results, including accuracy, recall, and F1-measure, show that the proposed model can fully integrate the weight of emotional feature words. It effectively improves the sentiment classification performance of imbalanced short-text review data.

## 1. Introduction

In the era of information and big data [1], massive data can be acquired, stored, and applied [2]. By mining the comment attitude in this information, it plays a certain role in the purchase decision of potential consumers [3]. Mining this review information [4] to identify people’s evaluation and attitudes toward products, services, and their attributes is a process called sentiment analysis (SA) [5]. In recent years, sentiment analysis based on deep-learning methods [6] has gradually become the mainstream. At present, the commonly used deep learning methods in sentiment analysis mainly include the convolutional neural network (CNN) [7,8], the long short-term memory network (LSTM) [9,10], and their variants.

The excellent performance of most methods is based on the assumption that the samples between classes in the dataset are balanced [11,12,13]. For example, Irsoy et al. [11] applied RNN for text sentiment orientation classification, Kim et al. [12] used CNN for text sentiment orientation classification, and Soni et al. [14] proposed TextConvoNet, a novel convolutional neural network (CNN)-based architecture for solving binary and multi-class text classification problems. At the same time, some scholars have begun to study the classification problem based on imbalanced data [15,16]. For example, Yin Hao et al. [17] proposed a resampling multi-channel model, which randomly sampled the imbalanced samples to make the number of samples balanced before training. Previous studies show that the use of deep learning methods can indeed be better for text classification, but these methods are mainly for text extraction or loss improvement and are based on the assumption of balanced sample distribution, and the research on sentiment classification methods for imbalanced data is still lacking. In response to the above problems, this paper proposes a sentiment classification method using a multi-channel BLTCN-BLSTM self-attention network combined with loss rebalancing and classification algorithm enhancement. The model in this paper is a comprehensive improvement method. Firstly, word embedding takes the processed sample as the input of the multi-channel, and then fuses the self-attention mechanism to strengthen the emotion to fully extract the text features. Finally, combined with comparative experiments and ablation experiments, the multi-channel BLTCN-BLSTM self-attention network of the proposed model improves the classification performance of imbalanced short texts to a certain extent.

Section 1 introduces the content of the short passage or related background. Section 2 introduces the related work. Section 3 provides the details of the model construction, where Section 3.1 describes the imbalanced short text sentiment classification modeling process, Section 3.2 describes the model vector embedding layer, Section 3.3 describes the multi-channel BLTCN-BLSTM self-attention network model, Section 3.4 describes the L-Softmax augmentation, and Section 3.5 presents the loss rebalancing. Then, Section 4 provides experiments and analysis, where Section 4.1 provides the experimental environment, Section 4.2 introduces the experimental data, Section 4.3 provides evaluation metrics, Section 4.4 performs an experimental comparison, Section 4.5 performs ablation experiments, and Section 4.6 performs predictive text self-attention visualization. Finally, Section 5 provides a conclusion.

## 2. Related Work

In the early years, sentiment dictionaries [18] and traditional machine learning [19] were mainly used, and Kamps et al. [20] used WordNet for sentiment classification. Compared with English dictionary research, the research on Chinese sentiment dictionary is relatively small. Dong Zhendong et al. [21] marked HowNet for sentiment classification, but the expansion was limited because the labeling process was time-consuming and laborious. Pang et al. [22] used the machine learning method of support vector machine (SVM) [23] to analyze the sentiment of text and found that their method could achieve the optimal effect. Lee et al. [24] applied the maximum entropy method to sentiment classification in electronic product reviews. These methods usually require suitable feature selection in the classification process, and thus lead to poor scalability.

In recent years, sentiment orientation classification has begun to use a deep learning framework to realize the automatic selection of features by using algorithms, so it has strong applicability in the field. Kim et al. [12] carried out text classification based on the method of CNN convolutional network, and extracted local key information by setting a variety of convolution kernels. Each word was a one-dimensional vector, and a sentence was composed of a matrix, and then the pooling was used to reduce the dimension, and finally, the sigmoid function was used for classification, but the context connection was not considered. Sitaula et al. [25] proposed an integrated feature extraction CNN model for emotion classification of NepCOV19Tweets. Lin et al. [26] proposed an advanced TF-IDF mechanism based on the CNN model to extract feature semantic information by considering word embedding coefficients. Irsoy et al. [11] applied RNN for text sentiment orientation classification. RNN recurrent neural networks better include the time sequence relationship of input information. Bai et al. [27] applied PG-RNN position-gated recurrent neural network to dynamically integrate global and local information. Xu et al. [28] applied an LSTM recurrent network to classify texts, and considered the order dependence between word orders by capturing the distance far and near, which effectively alleviated the gradient disappearance and gradient explosion problems faced by RNN, making LSTM widely used in various fields. Wang et al. [29] introduced the self-attention mechanism and aspect word embedding into the traditional LSTM model, which divides the sentiment of different aspects in a sentence. Soni et al. [30] proposed a hybrid model based on the fusion of long short-term memory and encoder for sentiment analysis. Abdi et al. [31] proposed SAS embedding space and a method based on deep learning, namely the RNN-LSTM algorithm. Although the above models effectively improve the prediction effect of the model, they do not integrate the advantages of CNN and LSTM in extracting text features, and most of them assume that the sample data between classes are balanced. The imbalanced distribution of sample data has brought great challenges to the training of neural networks.

Scholars have also conducted many studies on the coexistence of sentiment classification and class imbalance in text. Ye et al. [32] improved the recognition accuracy of the minority class by performing multiple k-means clustering on the majority class. Yin Hao et al. [17] proposed a multi-channel LSTM model for data resampling, and the imbalanced number was resampled, so that multiple groups of data were input into the multi-channel LSTM network for sentiment classification. Xiao et al. [33] first trained the CNN model on the balanced data, transferred the trained model to the imbalanced dataset, and undersampled the imbalanced dataset to make the data balanced. Cao et al. [34] proposed a method based on margin minimization loss to classify imbalanced samples, which replaced the traditional cross-entropy loss and added a training table to make the model postpone the update of loss weights and pay more attention to key information.

The above methods mainly consider loss improvement in text extraction or resampling, and do not take into account feature text feature extraction and loss improvement at the same time. This paper intends to improve these two aspects simultaneously. This paper proposes a sentiment classification method using a multi-channel BLTCN-BLSTM self-attention network combined with loss rebalancing and classification algorithm enhancement, which is a comprehensive improvement method. This method not only takes into account text feature extraction, but also combines the Focal Loss factor to reduce the loss caused by data imbalance, and proposes integrating L-Softmax into the text sentiment orientation binary classification task to improve the performance of text feature learning and classification.

## 3. Construction of the Model

### 3.1. Sentiment Classification Modeling for Imbalanced Short Texts

**Figure 1:**
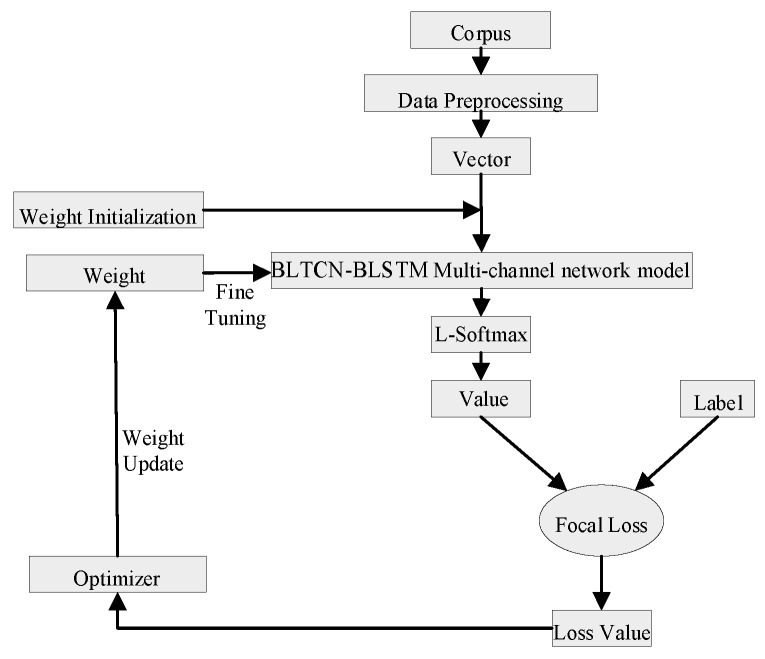
Multi-channel BLTCN-BLSTM self-attention model process.

The modeling of the model in this paper was divided into corpus processing, vectorized representation, feature extraction, sentiment classification, and other processes. As shown in Figure 1, the text was mapped to a vector representation. The text was composed of a position vector and character vector, and the feature extraction was performed by a multi-channel BLTCN-BLSTM self-attention network. The extracted fusion features were concatenated as the input of the fully connected layer, and finally, the emotion category was recognized by the L-Softmax classifier.

### 3.2. Vector Embedding Layer

The word vector input of the above studies was relatively single, and this paper intends to fuse word information and word position information as the input of the embedding layer. If the length of each sentence is m, when m is greater than max_len, the words with the length before max_len are intercepted. When m is less than max_len, the sentence is completed by adding zero at the end of the sentence. The proposed model maps each word in a sentence into a vector $x_{i}$; then, the sentence sequence can be represented as $X=[x_{1},x_{2},\ldots ,x_{k}]$, where each $x_{i}$ vector contains two parts of embedding sum, word vector encoding, and position vector encoding.

**Figure 2:**
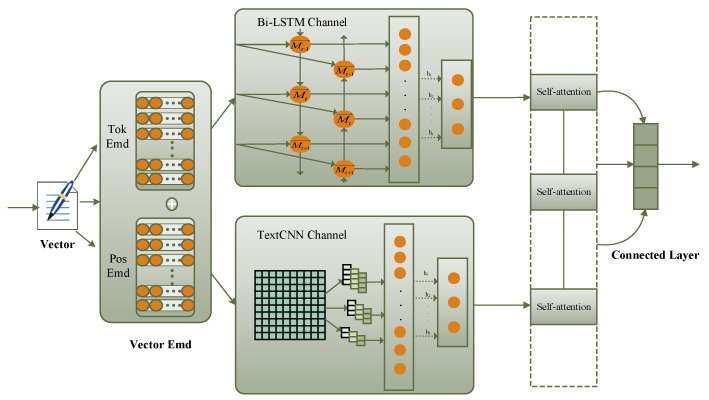
BLTCN-BLSTM multi-channel self-attention network model.

(1) Tok, in Figure 2, stands for word vector encoding. The words are vectorized to represent C, and each comment sentence is shown in (1) after being vectorized.

(1) $$C={\left[c_{1},c_{2},c_{3},\ldots ,c_{n}\right]}^{T}\in R^{m\times n}$$

Here, n represents the dimension of the word vector, m represents the number of words in each review, and $c_{i}$ represents the i-th word vector in the text $\left(i=1,2,3,\ldots ,n\right)$.

(2) Pos represents the current position encoding vector, and the position encoding dimension is consistent with the Tok word vector dimension. By adding Tok and the position encoding vector together, the added word has the word information with the position. The position vector is obtained by calculating formulas such as (2) and (3).

(2) $$\mathrm{PE}(\mathrm{pos},2i)=\sin(\mathrm{pos}/10000^{2i}/d_{n})$$

(3) $$\mathrm{PE}(\mathrm{pos},2i+1)=\cos(\mathrm{pos}/10000^{2i}/d_{n})$$

pos refers to the position of the word in the sentence. i is the position parameter, the even position of the word in the sentence is calculated using Formula (2), the cardinal position is calculated using Formula (3), and $d_{n}$ refers to the word vector dimension.

### 3.3. Multi-Channel BLTCN-BLSTM Self-Attention Network Model

After the vectorized representation of the text, it was input into channel one and channel two simultaneously. The multi-channel BLTCN-BLSTM self-attention network model is used to grasp multi-level and multi-dimensional semantic information. The TextCNN channel extracts different levels of feature expressions through three different convolution kernels. The Bi-LSTM bidirectional channel mainly solves the problem of timing and so on.

As shown in Figure 2, the multi-channel BLTCN-BLSTM self-attention model is composed of bidirectional dynamic encoding, convolution, cross-channel feature fusion layer, and self-attention layer.

(1) TextCNN channel: The TextCNN model applies the CNN model to the text. Its structure includes the input layer, multiple convolution layer, pooling layer, fully connected layer, and output layer. In processing text tasks, due to the special form of text, the way of convolution is generally one-dimensional convolution, and the width of the convolution kernel is consistent with the dimension of the word vector. The N-gram local features are extracted by a convolution operation. TextCNN channel takes the Vector embedding layer vector as the input of the convolution channel, and the dimension of the word vector is k. The convolution layer uses h sliding windows of different sizes to perform convolution operations on the text input vector to learn text features. The h convolution kernel sizes are 2, 3, 4, respectively. Each filter_size has a different channel, which is processed to obtain the regional feature map. After calculating the dimension reduction of each feature map by the Max pooling method through the pooling layer, the channel vectors are merged into a whole through the connection operation, which is used as the output of the TextCNN channel. The eigenvalues are obtained by the convolution kernel at position i, and the formula is as in (4).

(4) $$s=f(w\cdot T_{i:i+h-1}+b),w\in R^{h\times k}$$

**Figure A1:**
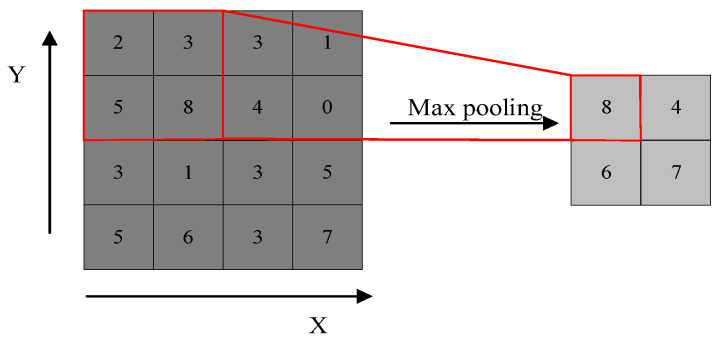
Maximum pooling process diagram.

Here, k represents the vector dimension corresponding to each word in the text sequence, w represents the convolution kernel with dimension size h×k, and $T_{i:i+h-1}$ represents the sliding window consisting of row i to row i+h-1 of the input matrix. b denotes the bias term parameter and f denotes the nonlinear mapping function. After the word set under each convolution window, the Max pooling method [35] is used to reduce the feature dimension by the vector–matrix pooling operation, as shown in Figure A1. The calculation formula is as follows: $Z_{i}=\max\left\{c_{i}\right\}$, where $c_{i}$ represents the feature vector obtained by convolution operation, and finally, the vector splicing is carried out.

**Figure A2:**
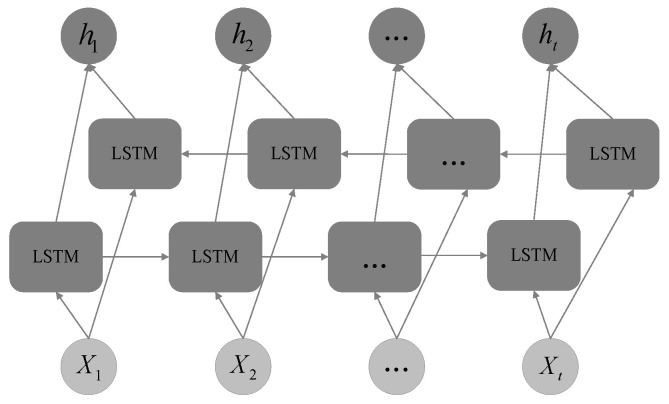
Bi-LSTM network structure diagram.

(2) Bi-LSTM bidirectional channel: Although the multi-channel TextCNN can effectively extract local features, it cannot consider the temporal features of the sentence at the same time. To make up for this defect, the LSTM long short-term memory network model was introduced. LSTM can be used to process the temporal information of sentences. By integrating the input information of the history unit and the current time unit, the “memory” unit that reflects the global information is generated after the operation. However, LSTM found the problem that information cannot be effectively transmitted in long-term experiments, and the main reason is that the results of semantic encoding by this network are biased toward the semantics corresponding to the last words in the text [36]. This problem can be effectively solved by designing a bidirectional memory network. The structure of Bi-LSTM is shown in Figure A2, and it is jointly trained by a forward and a backward, respectively, taking forward and backward information, as shown in Formulas (5)–(11).

(5) $$\vec{f_{t}}=\sigma (\vec{W_{f}}h_{t-1}+\vec{U_{f}}x_{t}+\vec{b_{f}})$$

(6) $$\vec{i_{t}}=\sigma (\vec{W_{i}}h_{t-1}+\vec{U_{i}}x_{t}+\vec{b_{i}})$$

(7) $$\tilde{c_{t}}=\tanh(\vec{W_{c}}h_{t-1}+\vec{U_{c}}x_{t}+\vec{b_{c}})$$

(8) $$\vec{c_{t}}=\vec{f_{t}}*\vec{c_{t-1}}+\vec{i_{t}}*\tilde{c_{t}}$$

(9) $$\vec{o_{i}}=\sigma (\vec{W}h_{t-1}+\vec{U}x_{t}+\vec{b})$$

(10) $$\vec{h_{t}}=\vec{o_{t}}*\tanh(\vec{c_{t}})$$

(11) $$\overset{\leftarrow }{h_{t}}=\overset{\leftarrow }{\mathrm{LSTM}}(x_{t}),t\in \left[1,T\right]$$

Here, $\vec{f_{t}}$, $\vec{i_{t}}$, and $\vec{o_{t}}$ denote the parts of the $\vec{\mathrm{LSTM}}$ forget gate, input gate, and output gate at time t forward, respectively. σ is the activation function sigmoid. $\tilde{c_{t}}$ is the input gate candidate cell. $\vec{c_{t}}$ represents the output of the forward memory control unit after updating at time t. $\vec{W}$ and $\vec{U}$ are the weight matrices of the forward class. $\vec{b}$ denotes the offset vector of the forward class. Backward $\overset{\leftarrow }{\mathrm{LSTM}}$, as in the formula, is the same as forward. $x_{t}$ represents the input vector, forward $\vec{h_{t}}$ is learned from $x_{1}$ to $x_{t}$ at time t, backward $\overset{\leftarrow }{h_{t}}$ is learned from $x_{t}$ to $x_{1}$ at time t, and $\vec{h_{t}}$ and $\overset{\leftarrow }{h_{t}}$ are concatenated to obtain the final hidden layer representation $h_{t}=\vec{h_{t}}⊕\overset{\leftarrow }{h_{t}}$.

(3) Self-attention layer: Self-attention is a variant of attention called internal attention. Its advantage is that it can directly calculate the dependencies between vectors without external additional information, which can better learn the internal structure of sentences. The vector H output from the CNN channel and Bi-LSTM bidirectional channel is input into the self-attention layer to learn the dependencies within the sequence and the weights of different word vectors.

(12) $$S_{self-attention}=\mathrm{softmax}(\frac{H\cdot H^{Τ}}{\sqrt{d_{k}}})H$$

where H is the input information of the self-attention layer; then, the scaled dot product operation is used, $d_{k}$ is the dimension of the embedded word vector, and the calculation formula is as shown in (12).

**Figure 3:**
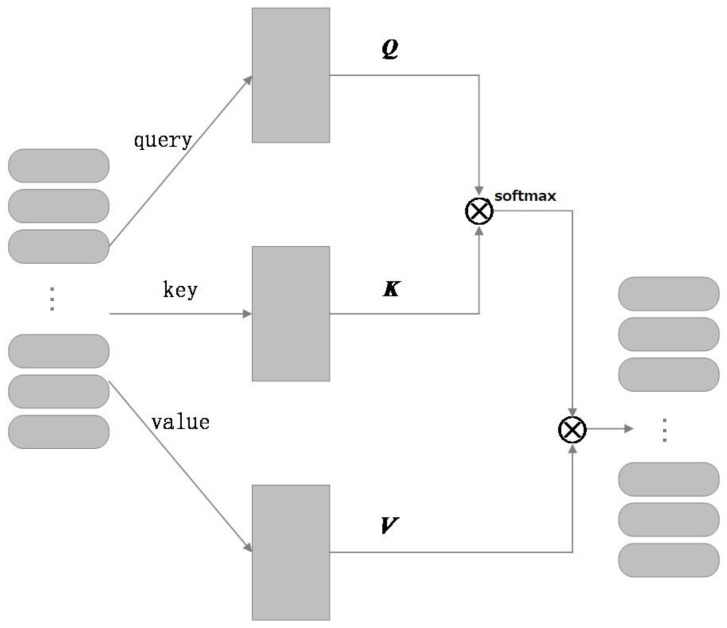
Self-attention mechanism structure.

The structure of the self-attention mechanism is shown in Figure 3. The self-attention mechanism usually adopts the Query-Key-Value (QKV) method, where the source of Q, K, and V is the same input. Let the input matrix be H, and the matrices Q, K, and V are obtained by different matrix transformations. Firstly, the similarity is calculated by the transpose multiplication of Q matrix and K matrix, and the obtained matrix is input into softmax for normalization. Finally, the normalized matrix is multiplied by the V matrix to obtain the calculation results of the self-attention layer.

(4) Connected layer: The information fusion operation is adopted for the high-level features that pass through multiple channels to obtain the fused text feature representation D. The calculation formula is as follows (13).

(13) D=BL⊕TC

BL is the text feature vector output by the Bi-LSTM bidirectional channel after attention, TC is the text feature vector output by the TextCNN channel after attention, and the fused text feature vector D is classified by the L-Softmax classifier.

By constructing the multi-channel BLTCN-BLSTM network, respectively, the text features are extracted by combining semantic knowledge, which solves the local feature extraction, and effectively ensures the distance dependence and the correlation between attributes. Then, the learned feature vectors are given different weights through the self-attention layer to strengthen the learning of emotional semantic features.

### 3.4. L-Softmax Augmentation

The output vector after dimensionality reduction of the above channels was inputted into the L-Softmax layer. To solve the problem of feature learning classification performance degradation caused by imbalanced samples, this model fuses L-Softmax, adds an Angle constraint in feature learning, and adds a hyperparameter n representing the class interval to make the boundary of different categories of samples more obvious. This study found that the classification effect of L-Softmax was better than Softmax when n=2 was used in this model. The calculation formula is as in (14).

(14) $$\left\|w_{1}^{T}\right\|\cdot \left\|x\right\|\cos\left(\theta_{1}\right)+b_{1}>\left\|w_{1}^{T}\right\|\cdot \left\|x\right\|\cos\left(n\theta_{1}\right)+b_{1}>\left\|w_{2}^{T}\right\|\cdot \left\|x\right\|\cos\left(\theta_{2}\right)+b_{2}$$

For the parameter $0\leq \theta_{1}\leq \frac{\pi }{n}$, L-Softmax puts forward higher requirements in the process of learning weights $w_{1}$ and $w_{2}$, so that there is a wider decision boundary between feature learning classes.

### 3.5. Loss Rebalancing

After the L-Softmax layer, Focal Loss was fused again. By reducing the contribution value to the loss of easy text samples and increasing the contribution value to the loss of difficult samples, the optimal parameters were found to solve the model performance problems caused by the imbalance of overall data categories. The calculation formula is given in (15) and (16).

(15) $$p_{t}=\left\{\begin{array}{cc} p & \mathrm{if}\begin{array}{c} y=1 \end{array}\\ 1-p & \mathrm{otherwise} \end{array}\right.$$

(16) $$FL(p_{t})=-\alpha {\left(1-p_{t}\right)}^{\gamma }\log(p_{t})$$

where $p_{t}$ represents the probability that the sample is predicted to be a certain class, y represents the class label of the sample, the weighting factor α∈[0,1] can balance the weight of the difference between the number of positive and negative samples on the total loss, $1-p_{t}$ is the modulation factor, and the focusing parameter γ>0 reduces the loss of easily divided samples. Focal Loss algorithm, when the sample is correctly classified, the larger $p_{t}$ is, the higher the classification confidence is, indicating that the sample is easy to classify. The smaller $p_{t}$ is, the lower the classification confidence is, which means the sample is difficult to distinguish. The experiment found that when the parameters were α=0.25 and γ=2, the experimental effect was the best; for example, if $p_{t}=0.8$, then $FL(p_{t})=0.00097$, and this sample easily distinguishes that the loss value contribution decreases; if $p_{t}=0.1$, then $FL(p_{t})=0.20250$, and it is hard to distinguish that the loss value contribution increases in this sample.

## 4. Experiment and Analysis

### 4.1. Experimental Environment

In the deep learning model training experiment, parameter settings will have a great impact on the results. After experimental comparison, the experimental parameters in this paper are shown in Table 1 and Table 2. The batch_size of the TextCNN channel and Bi-LSTM bidirectional channel is 32.

### 4.2. Experimental Data

The pre-trained model used in this experiment is the Chinese open-source pre-training model “bert-base-chinese” by HIT (Harbin Institute of Technology) [37]. The data used are the ChnSentiCorp-Htl- 10,000 dataset [38]. ChnSentiCorp-Htl- 10,000 contains a total of 3000 negative comment samples and 7000 positive comment samples, and the sample totals 10,000 corpora. For repeated samples and other processing, the number of negative and positive samples are 2443 and 5322, respectively. The sample of data after removing special characters is shown in Table 3. Each comment in the dataset was artificially set with a sentiment label, and the comment set is dominated by short texts. The experimental dataset was randomly divided into training and testing sets with an 8:2 ratio. The 5-fold cross-validation was set up, and the average was taken.

**Figure 4:**
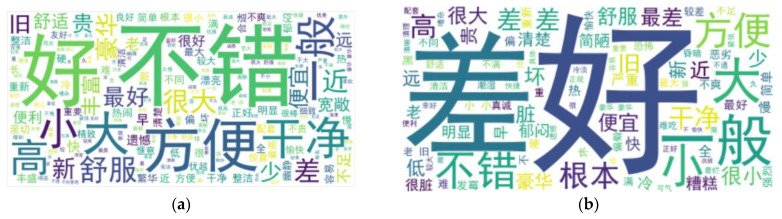
(**a**) Positive word cloud map; (**b**) negative word cloud map.

The processed positive and negative samples can be seen by plotting the word cloud in Figure 4. Positive samples have more positive evaluation words, such as “好(nice)” and “方便(convenient)”, while negative samples have more negative evaluation words, such as “糟糕(worst)” and “差(terrible)”.

### 4.3. Evaluation Index

The classification evaluation criteria [39] used in the experimental model of this study are accuracy ($V_{\mathrm{accuracy}}$), precision ($V_{\mathrm{precision}}$), recall ($V_{\mathrm{recall}}$), and $F_{1}$-measure of classification, as shown in Table 4.

The specific calculation formula is as follows: (17)–(20).

(17) $$V_{\mathrm{accuracy}}=\frac{\mathrm{TP}+\mathrm{TN}}{\mathrm{TP}+\mathrm{FN}+\mathrm{FP}+\mathrm{TN}}$$

(18) $$V_{\mathrm{precision}}=\frac{\mathrm{TP}}{\mathrm{TP}+\mathrm{FP}}$$

(19) $$V_{\mathrm{recall}}=\frac{\mathrm{TP}}{\mathrm{TP}+\mathrm{FN}}$$

(20) $$F_{1}=\frac{{2V}_{\mathrm{precision}}\times V_{\mathrm{recall}}}{V_{\mathrm{precision}}+V_{\mathrm{recall}}}$$

### 4.4. Experimental Comparison

In order to verify the classification performance of the proposed model, the multi-channel BLTCN-BLSTM self-attention network model proposed in this paper was compared with the other 10 neural network models on the dataset ChnSentiCorp-Htl. The experimental Settings are as follows, and the experimental results are shown in Table 5.

(1) RNN [13]: The word embedding settings were unchanged; only the RNN single-channel model was used, L-Softmax enhancement was not added, and Focal Loss was rebalanced.

(2) TextCNN [14]: The word embedding settings were unchanged; only the TextCNN single-channel model was used, L-Softmax enhancement was not added, and Focal Loss was rebalanced.

(3) LSTM [28]: The word embedding settings were unchanged; only the LSTM single-channel model was used, L-Softmax enhancement was not added, and Focal Loss was rebalanced.

(4) BiLSTM [17]: The word embedding settings were unchanged; only the BiLSTM single-channel model was used, L-Softmax enhancement was not added, and Focal Loss was rebalanced.

(5) TextCNN_BiLSTM: The word embedding settings were unchanged, TextCNN_BiLSTM multi-channel model and L-Softmax enhancement were not added, and Focal Loss was rebalanced.

(6) RNN-FL-LS: The word embedding settings were unchanged; only the RNN single-channel model and L-Softmax enhancement were added, and Focal Loss rebalancing was performed. 

(7) TextCNN-FL-LS: The word embedding settings were unchanged; only the TextCNN single-channel model was used, L-Softmax enhancement was added, and Focal Loss was rebalanced. 

(8) LSTM-FL-LS: The word embedding settings were unchanged; only the LSTM single-channel model was used, L-Softmax enhancement was added, and Focal Loss was rebalanced. 

(9) BiLSTM-FL-LS: The word embedding settings were unchanged; only the BiLSTM single-channel model was used, L-Softmax enhancement was added, and Focal Loss was rebalanced. 

(10) TextCNN_BiLSTM-FL-LS: The word embedding settings were unchanged, the TextCNN_BiLSTM multi-channel model was added, L-Softmax enhancement was added, and the Focal Loss was rebalanced. 

(11) BLTCN-BLSTM: The word embedding settings were unchanged, and the multi-channel self-attention model proposed in this paper was enhanced by L-Softmax and rebalanced by Focal Loss.

Accuracy ($V_{\mathrm{accuracy}}$), precision ($V_{\mathrm{precision}}$), recall ($V_{\mathrm{precision}}$), and F1-measure in Table 4 are used as evaluation criteria.

As can be seen from Table 5, the F1 value of the BiLSTM model is 0.4% higher than that of the LSTM model, indicating that the bidirectional LSTM performs better than the unidirectional LSTM in temporal feature extraction. The model BLTCN-BLSTM in this paper achieves the best sentiment classification effect on the experimental dataset, and the accuracy, recall, and F1-measure are 89.1%, 89.2%, and 89.3%, respectively, which are higher than other models. The F1 value of the TextCNN-bilSTM model is 88.3%, which is 4.3% and 1.1% higher than that of only using TextCNN and only using BiLSTM. It is verified that the combination of multi-channel can extract local information and temporal features at the same time, which is conducive to the effect of imbalanced text sentiment classification. By adding FL-LS to RNN, TextCNN, LSTM, BiLSTM, and TextCNN-bilST, the classification F1 value of RNN, TextCNN, LSTM, and TextCNN-bilST is increased by 0.4%, 0.5%, 0.4%, 0.5%, and 0.6%, respectively, compared with that without adding FL-LS. It shows that, on the one hand, the classification effect of imbalanced text can be increased by feature extraction enhancement, and the classification effect can also be improved by the loss rebalancing level and the decision level. By analyzing the experimental results, it can be seen that using multi-channel to extract the features of imbalanced text, the results not only contain information about the features themselves, but also contain the relationship between the features. Through the features of different channels, the mutual connections between different features and more hidden information are fully mined, which effectively makes up for the defects that it is difficult to extract more feature information by using a single channel, so as to improve the classification effect of the imbalanced text.

### 4.5. Ablation Experiment

To verify the comprehensive improvement in the proposed method in all aspects, five kinds of ablation experiments were carried out below. The models compared were Att-TCN-BLSTM, ATT-TCN-BLSTM-FL, ATT-TCN-BLSTM-LS, and BLTCN-BLSTM. The experimental setup is as follows, and Table 6 presents the results of the ablation experiments.

(1) TCN-BLSTM: The word embedding settings were unchanged, and no self-attention layer was added to the multi-channel.

(2) Att-TCN-BLSTM: The word embedding settings were unchanged, and multi-channel self-attention layers were added.

(3) Att-TCN-BLSTM-FL: The word embedding settings were unchanged, the self-attention layer was added to the multi-channel, and L-Softmax enhancement was added. 

(4) Att-TCN-BLSTM-LS: The word embedding settings were unchanged, the self-attention layer was added to the multi-channel, and the Focal Loss was added for rebalancing. 

(5) BLTCN-BLSTM: The word embedding settings were unchanged for the proposed model.

The experiments show that when the self-attention layer and FL-LS improvement are removed, the F1 value of the proposed model is reduced by 1.0%. The F1 value of the proposed model is reduced by 0.8% when only adding the self-attention mechanism layer and removing FL-LS. The F1 value of the proposed model is reduced by 0.6% when only LS is removed. The F1 value of the proposed model is reduced by 0.9% when only FL is removed. In summary, after adding the self-attention layer, loss rebalancing factor, and decision interface optimization, the classification effect of the imbalanced short text of the proposed model is improved, and all aspects have effects.

### 4.6. Predictive Text Self-Attention Visualization

To visually and effectively show the effect of the model after self-attention, this paper uses a heat map to visualize the sentence attention weight allocation in the predicted text. The prediction text selects one paragraph for each positive and negative review.

Text 1: 总体感觉还不错，房间很干净、交通方便。 (“The overall feeling is good, the room is very clean, convenient transportation.”)

Text 2: 酒店前台的小孩服务态度实在太差。 (“The child service attitude at the hotel front desk is terrible.”)

**Figure 5:**
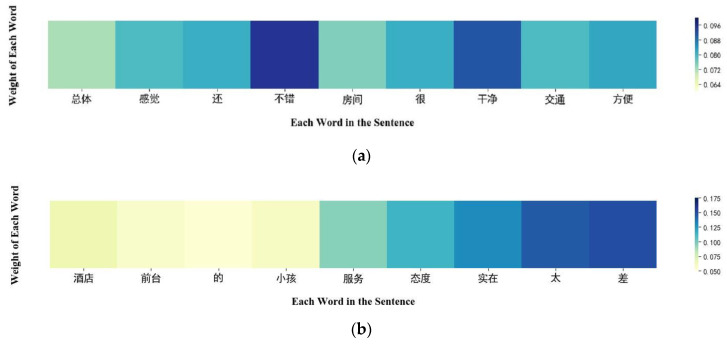
(**a**) Text 1 attention weight heatmap(“总体 (The overall)” “感觉 (feeling)” “还 (still)” “不错 (good)” “房间 (room)” “很 (very)” “干净 (clean)” “交通 (traffic)” “方便 (convenient)”); (**b**) Text 2 attention weight heatmap(“酒店 (The hotel)” “前台 (front desk)” “的 (the)” “小孩 (child)” “服务 (service)” “态度 (attitude)” “实在 (reality)” “太 (too)” “差 (terrible)”).

For two prediction texts, heat maps of word-level attention weights were drawn. The darker the color in the figure, the larger the gray value, which means the higher the weight of the assigned word, the more important the word is for the sentiment classification of the sentence, and vice versa. The load marker uses “bert-base-chinese” pre-trained model parameters. Prediction Text 1 Figure 5 shows that the model assigns high weights to words such as “不错 (nice)” and “干净 (clean)”, which are biased toward positive reviews. Prediction Text 2 Figure 5 shows that the model assigns high weights to the words “太 (too)” and “差 (poor)”, which are biased toward negative comments.

Through the above visual analysis of the predicted text self-attention weight, it shows that this paper can use the multi-channel self-attention mechanism to find the words that have a great impact on the sentiment classification results in the sentence. It proves that the proposed model can indeed pay more attention to the more important sentiment words in the sentence.

## 5. Conclusions

In summary, this paper combines the advantages of CNN and LSTM in text processing to construct a multi-channel BLTCN-BLSTM self-attention network model. The self-attention mechanism is integrated into the text features extracted by multi-channel BLTCN-BLSTM at the same time to enhance the ability of the model to capture key semantic information. The Focal Loss function is integrated from the loss level to reduce the difference in model classification performance caused by an imbalanced corpus in deep learning, and the L-Softmax hyperparameter factor is introduced from the decision classification level to further improve the classification effect of the decision interface. Combined with comparative experiments and ablation experiments, the performance of the proposed model method is verified; that is, the problem of emotional binary classification of imbalanced short texts is alleviated. However, there are limitations to text collection. Through the research of this paper, this method can be applied to multi-classifier and multi-language sentiment classification in the future to further improve the effect of imbalanced text sentiment classification.

## Figures and Tables

**Table 1:** TextCNN Channel model hyperparameter setting table.

Hyper Parameter	Value
Size of Word Vector	128
Filter_Size	(2,3,4)
Epochs	10
Learning Rate of Adam	0.001
Pooling	Max Pooling

**Table 2:** Bi-LSTM bidirectional channel model super parameter setting table.

Hyper Parameter	Value
Size of Word Vector	128
Epochs	10
Hidden_Size	128
Drop_out	0.5
Activation Function	RuLu

**Table 3:** Example Dataset.

Comments	Label
房间设施远达不到四星水平，勉强也就是三星。(“Room facilities are far from four stars, barely three.”)	Neg
这个酒店，隔音太差，纯净饮用水坏了，也不配给免费矿泉水。(“In this hotel, the sound insulation is too poor, the pure drinking water is bad, and the free mineral water is not rationed.”)	Neg
从价格来说，性价比很高168元，前台服务员态度很好。(“From the price, cost-effective 168 yuan, the front desk staff attitude is very good.”)	Pos
很不错的酒店，尽管在火车站，但很安静，房间干净。(“It’s a nice hotel, even though it’s at the train station, it’s quiet and the rooms are clean.”)	Pos

**Table 4:** Confusion matrix of classification results.

	Value Positive	Negative
True	TP (True Positive)	TN (True Negative)
False	FP (False Positive)	FN (False Negative)

**Table 5:** Comparison of experimental results of different models.

Model	ChnSentiCorp-Htl- 10,000			
	Accuracy	Precision	Recall	F1-Measure
RNN	0.852	0.850	0.856	0.853
TextCNN	0.841	0.838	0.842	0.840
LSTM	0.865	0.867	0.869	0.868
BiLSTM	0.874	0.871	0.873	0.872
TextCNN-BiLSTM	0.882	0.886	0.880	0.883
RNN-FL-LS	0.856	0.855	0.859	0.857
TextCNN-FL-LS	0.846	0.843	0.847	0.845
LSTM-FL-LS	0.869	0.871	0.873	0.872
BiLSTM-FL-LS	0.879	0.876	0.878	0.877
TextCNN-BiLSTM-FL-LS	0.887	0.892	0.886	0.889
BLTCN-BLSTM	0.891	0.894	0.892	0.893

**Table 6:** Comparison of the ablation results of the models.

Model	ChnSentiCorp-Htl- 10,000			
	Accuracy	Precision	Recall	F1-Measure
TCN-BLSTM	0.882	0.886	0.880	0.883
Att-TCN-BLSTM	0.884	0.888	0.882	0.885
Att-TCN-BLSTM-FL	0.886	0.889	0.885	0.887
Att-TCN-BLSTM-LS	0.885	0.882	0.886	0.884
BLTCN-BLSTM	0.891	0.894	0.892	0.893

## Data Availability

Not applicable.
